# Functional Comparison of High and Low Molecular Weight Chitosan on Lipid Metabolism and Signals in High-Fat Diet-Fed Rats

**DOI:** 10.3390/md16080251

**Published:** 2018-07-29

**Authors:** Shing-Hwa Liu, Chen-Yuan Chiu, Ching-Ming Shi, Meng-Tsan Chiang

**Affiliations:** 1 Graduate Institute of Toxicology, College of Medicine, National Taiwan University, Taipei 100, Taiwan; shinghwaliu@ntu.edu.tw; 2 Department of Pediatrics, College of Medicine and Hospital, National Taiwan University, Taipei 100, Taiwan; 3 Department of Medical Research, China Medical University Hospital, China Medical University, Taichung 404, Taiwan; 4 Institute of Food Safety and Health, College of Public Health, National Taiwan University, Taipei 100, Taiwan; chenyuanchiu@ntu.edu.tw; 5 Department of Food Science, College of Life Science, National Taiwan Ocean University, Keelung 202, Taiwan; ni300ke6012@gmail.com

**Keywords:** high and low molecular weight chitosan, lipid metabolism, liver lipid accumulation

## Abstract

The present study examined and compared the effects of low- and high-molecular weight (MW) chitosan, a nutraceutical, on lipid metabolism in the intestine and liver of high-fat (HF) diet-fed rats. High-MW chitosan as well as low-MW chitosan decreased liver weight, elongated the small intestine, improved the dysregulation of blood lipids and liver fat accumulation, and increased fecal lipid excretion in rats fed with HF diets. Supplementation of both high- and low-MW chitosan markedly inhibited the suppressed phosphorylated adenosine monophosphate (AMP)-activated protein kinase-α (AMPKα) and peroxisome proliferator-activated receptor-α (PPARα) protein expressions, and the increased lipogenesis/cholesterogenesis-associated protein expressions [peroxisome proliferator-activated receptor-γ (PPARγ), sterol regulatory element binding protein-1c and -2 (SREBP1c and SREBP2)] and the suppressed apolipoprotein E (ApoE) and microsomal triglyceride transfer protein (MTTP) protein expressions in the livers of rats fed with HF diets. Supplementation with both a low- and high-MW chitosan could also suppress the increased MTTP protein expression and the decreased angiopoietin-like protein-4 (Angptl4) expression in the intestines of rats fed with HF diets. In comparison between low- and high-MW chitosan, high-MW chitosan exhibits a higher efficiency than low-MW chitosan on the inhibition of intestinal lipid absorption and an increase of hepatic fatty acid oxidation, which can improve liver lipid biosynthesis and accumulation.

## 1. Introduction

People who eat a Western pattern of diet, which is generally characterized by high calories, high protein, high fat and high salt, and have a low physical activity level tend to become obese and increase the risk of suffering the metabolic syndrome [1]. Obesity is one of the risk factors for the occurrence of cancer, cardiovascular disease, and diabetes. There are 39% of women and 39% of men aged ≥18 overweight and 18% of overweight or obese children and adolescents in 2016 [2]. Insulin resistance and dyslipidemia induced by obesity and diabetes are risk factors for the occurrence of nonalcoholic fatty liver disease (NAFLD) [3,4]. How to prevent and improve obesity and diabetes has become an important issue to reduce the prevalence of NAFLD.

Supplementation of chitosan has been reported to be responsible for regulating the metabolism of carbohydrates and lipids. Chitosan could reduce the activity of intestinal disaccharides, increase the excretion of lipids, improve insulin resistance, and prevent hepatic lipid accumulation in diabetic animal models [5,6,7,8]. Zeng et al. (2008) have shown that the influence of the absorption and distribution of chitosan in mice with oral administration is attributable to its water solubility and molecular weight (MW); the decreased MW and increased water solubility can enhance the absorption of chitosan molecules [9]. Chiu et al. (2017) have also discovered that low-MW chitosan produces greater effects than chitosan oligosaccharide to alleviate the abnormal intestinal disaccharidase activity and lipid metabolism in high-fat (HF) diet-fed rats [5]. Kondo et al. (2000) have shown that low-MW chitosan can prevent the progression of non-insulin-dependent diabetes mellitus by using low-dose streptozotocin (STZ) [10]. Yao et al. (2008) have found that high-MW chitosan possessed more potential than low-MW chitosan in decreasing hyperglycemia and hypercholesterolemia in diabetic rats [11]. Both low- and high-MW chitosan have been suggested to be capable of decreasing liver gluconeogenesis and increasing muscle glucose uptake to alleviate hyperglycemic effects in STZ-induced type 1 diabetic rats [7]. The regulation of both low- and high-MW chitosan in intestinal and liver lipid metabolism still remains to be further investigated. The present study examined and compared the effects of low- and high-MW chitosan on intestinal and hepatic lipometabolism in HF diet-fed rats. This study focuses on the ability of comparatively low- and high-MW chitosan to inhibit intestinal lipid metabolism and explore the differences of the effects of low- and high-MW chitosan on fatty liver in HF diet-fed rats.

## 2. Results

### 2.1. Effects of Low- and High-Mw Chitosan on Plasma Biochemical Indices, Organ Weight, and Body Weight in Hf Diet-Fed Rats

After 8 weeks of feeding different diets, the changes of food intake, caloric intake, body weight, and organ weight are shown in Table 1. The food intake was not affected in HF diet-fed rats with or without chitosan as compared to control rats. No behavioural alteration was observed by adding high-MW or low-MW chitosan to HF diet-fed rats. The final body weight was markedly higher in the HF diet-fed group than in the NC group, which could not be reversed by both low- and high-MW chitosan supplementations. Both low- and high-MW chitosan supplementations significantly inhibited the increased liver weight in HF diet-fed rats (Table 1). The relative adipose tissue weight showed no significant difference between the HF diet-fed + low- or high-MW chitosan group and the HF diet-fed group, although high-MW chitosan could reduce the weight of peripheral adipose tissue weight compared to the normal control group (Table 1). Both low- and high-MW chitosan supplementations significantly prolonged the length of the small intestine compared to the normal control group (Table 1).

Changes in the levels of blood lipids and tumor necrosis factor-α (TNFα) are shown in Table 2. The blood levels of total cholesterol (TC), low density lipoprotein-cholesterol (LDL-C) + very low density lipoprotein-cholesterol (VLDL-C), VLDL-C, LDL-C, high density lipoprotein-cholesterol (HDL-C), TC/HDL-C, and TNF-α were significantly upregulated in the HF diet group, which could be significantly reversed by both low- and high-MW chitosan supplementations. Unexpectedly, the blood triglyceride (TG) level was decreased in the HF diet group compared to NC group (Table 2). Both low- and high-MW chitosan supplementations markedly inhibited the HF diet group and decreased blood TG level, but there was no statistically significant difference compared to the NC group (Table 2). Moreover, the blood levels of liver function markers AST and ALT were markedly upregulated in the HF diet group, which could be significantly reversed by both high- and low-MW chitosan supplementations (Table 2).

### 2.2. Effects of Low- and High-Mw Chitosan on The Lipometabolism in Adipose and Liver Tissues and Feces of Hf Diet-Fed Rats 

**Figure 1:**
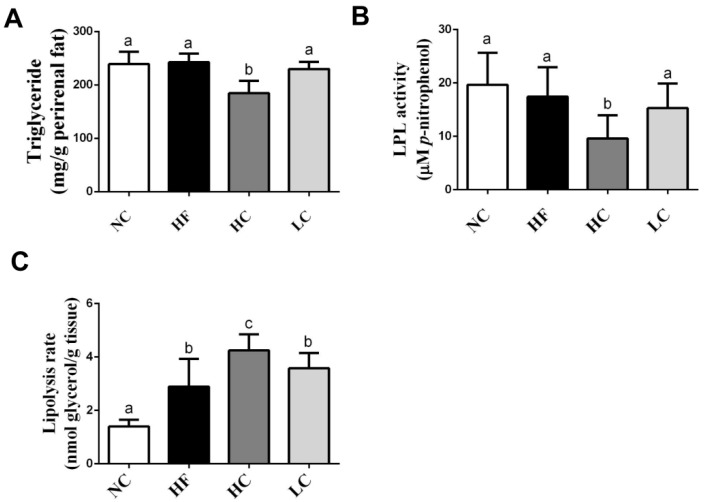
Effects of high- or low-MW chitosan on TG (**A**), lipoprotein lipase (LPL) (**B**), and lipolysis rate (**C**) in perirenal adipose tissues of HF diet-fed rats. The rats were fed with different experimental diets for 8 weeks. Data are presented as mean ± SD (*n* = 8). Different letters indicate significant differences (*p* < 0.05). NC, normal control +5% cellulose; HF, high fat diet +5% cellulose; HC, high fat diet +5% high-MW chitosan; LC, high fat diet +5% low-MW chitosan.

As shown in Figure 1A,B, the TG level and lipoprotein lipase (LPL) activity in the perirenal adipose tissues were significantly lower in the high-MW, but not low-MW, chitosan supplementation group than in the HF diet group, although there were no significant changes in the HF diet group compared to the NC group. High-MW, but not low-MW, chitosan supplementation could also significantly increase the lipolysis rate compared to the HF diet group (Figure 1C).

**Figure 2:**
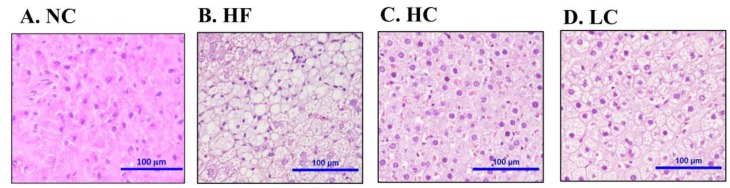
Effect of high- or low-MW chitosan on hepatic morphology in HF diet-fed rats. The livers were isolated from rats fed with different experimental diets for 8 weeks. Tissue sections were stained with hematoxylin and eosin. **A**: NC, normal control +5% cellulose; **B**: HF, high fat diet +5% cellulose; **C**: HC, high fat diet +5% high-MW chitosan; **D**: LC, high fat diet +5% low-MW chitosan.

We next investigated the effects of chitosan on lipid-related profiles in the livers of HF diet-fed rats. As shown in Table 3, there was severe TC and TG accumulation in the livers of HF diet-fed rats, which could be exhibited by a significant reversed effect by both low- and high-MW chitosan supplementations. Moreover, the histological examination showed severe hepatic vacuolization in HF diet-fed rats, which could be exhibited by a significant reversed effect by both low- and high-MW chitosan supplementations (Figure 2).

The fecal lipid-related profiles were also tested and shown in Table 4. Both low- and high-MW chitosan supplementations significantly upregulated the fecal weights and fecal TC and TG levels compared to the HF diet group.

### 2.3. Effects of Low- and High-Mw Chitosan on Lipometabolic Signals in the Liver, Blood, and Intestine of Hf Diet-Fed Rats

**Figure 3:**
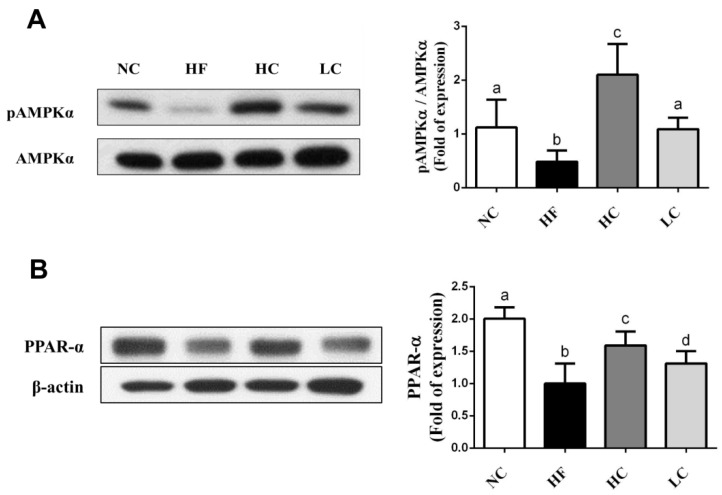
Effect of high- or low- MW chitosan on hepatic AMPKα and PPARα protein expressions in HF diet-fed rats. The rats were fed with different experimental diets for 8 weeks. Protein expressions of phosphorylated AMPKα/AMPKα (**A**) and PPARα (**B**) were determined by Western blot. Densitometric analysis for protein levels corrected to AMPKα or β-actin (internal control) was shown. Data are presented as mean ± SD (*n* = 6). Different letters indicate significant differences (*p* < 0.05). NC, normal control +5% cellulose; HF, high fat diet +5% cellulose; HC, high fat diet +5% high-MW chitosan; LC, high fat diet +5% low-MW chitosan.

**Figure 4:**
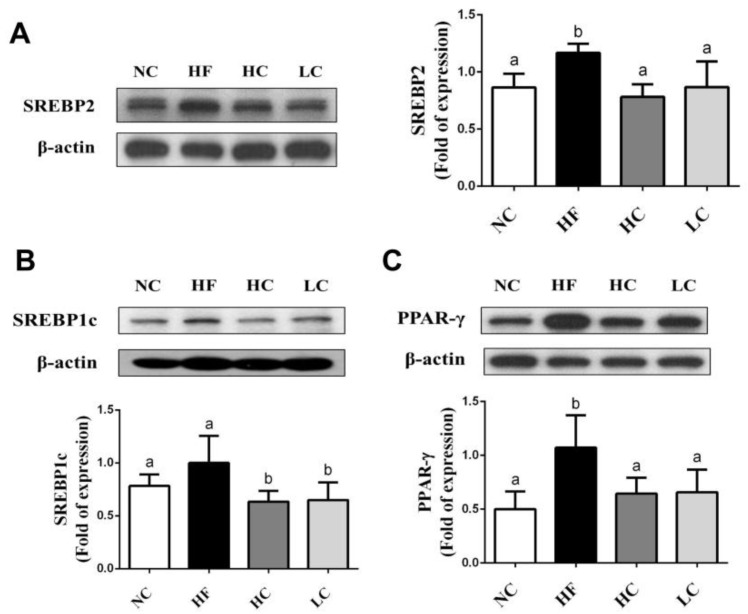
Effect of high- or low-MW chitosan on hepatic SREBP2, SREBP1c and PPARγ protein expressions in HF diet-fed rats. The rats were fed with different experimental diets for 8 weeks. Protein expressions of SREBP2 (**A**), SREBP1c (**B**), and PPARγ (**C**) were determined by Western blot. Densitometric analysis for protein levels corrected to β-actin (internal control) was shown. Data are presented as mean ± SD (*n* = 6). Different letters indicate significant differences (*p* < 0.05). NC, normal control + 5% cellulose; HF, high fat diet + 5% cellulose; HC, high fat diet + 5% high-MW chitosan; LC, high fat diet + 5% low-MW chitosan.

**Figure 5:**
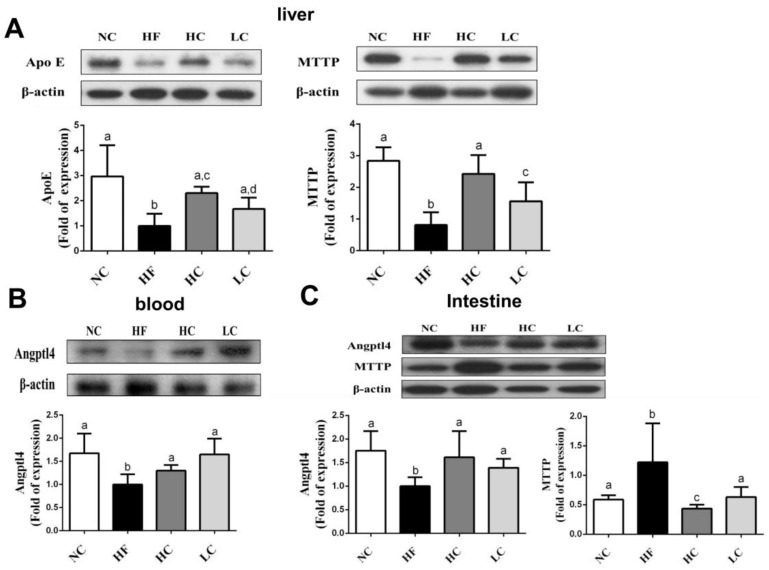
Effect of high- or low-MW chitosan on MTTP, ApoE, and Angptl4 protein expressions in the liver, blood, or intestine of HF diet-fed rats. The rats were fed with different experimental diets for 8 weeks. Protein expressions of hepatic ApoE and MTTP (**A**), blood Angptl4 (**B**), and intestinal MTTP and Angptl4 (**C**) were determined by Western blot. Densitometric analysis for protein levels corrected to β-actin (internal control) was shown. Data are presented as mean ± SD (*n* = 6). Different letters indicate significant differences (*p* < 0.05). NC, normal control +5% cellulose; HF, high fat diet +5% cellulose; HC, high fat diet +5% high-MW chitosan; LC, high fat diet +5% low-MW chitosan.

To evaluate the mechanisms of the preventive effects of low- and high-MW chitosan on HF diet-diversified homeostasis of lipids, we investigated the protein or gene expressions of lipometabolic regulators [adenosine monophosphate (AMP)-activated protein kinase-α (AMPKα), peroxisome proliferator-activated receptor-α (PPARα)], peroxisome proliferator-activated receptor-γ (PPARγ), sterol regulatory element binding protein-1c and -2 (SREBP1c and SREBP2)] [12] and lipid transport-related proteins [angiopoietin-like protein-4 (Angptl4), microsomal triglyceride transfer protein (MTTP), and apolipoprotein E (ApoE)] [13,14,15]. As shown in Figure 3, HF diet-fed rats markedly inhibited the protein expressions of PPARα and phosphorylated AMPKα (decreased pAMPKα/AMPKα ratio) in the liver, which could be exhibited by a significant reversed effect by both low- and high-MW chitosan supplementations. The effects of high-MW chitosan on AMPK phosphorylation and PPARα protein expression were significantly higher than low-MW chitosan (Figure 3). Moreover, both low- and high-MW chitosan supplementations significantly inhibited the increased protein expressions of SREBP2, SREBP1c, and PPARγ, in the livers of HF diet-fed rats (Figure 4). The effects of chitosan on the expressions of Angptl4, ApoE, and MTTP proteins were shown in Figure 5. HF diet feeding significantly decreased the protein expressions of hepatic MTTP, ApoE, plasma Angptl4, and intestinal Angptl4, and increased the protein expression of intestinal MTTP, which could be exhibited by a significant reversed effect by both low- and high-MW chitosan supplementations. The effects of high-MW chitosan on MTTP, ApoE and Angptl4 protein expressions were significantly better than low-MW chitosan (Figure 5).

## 3. Discussion

Absorption of chitosan in the intestine is mainly affected by the molecular weight and water solubility of chitosan. Low-MW chitosan is more easily absorbed in the intestine than high-MW chitosan [9]. The absorbed chitosan can be rapidly distributed from circulation to liver and kidney. The intestinal absorption and tissue distribution may be the factors to affect the healthy activities of chitosan. Sugano et al. (1980) showed that chitosan (200 kDa) supplementation possesses the potential to lower the hypercholesterolemic effect in HF diet-fed rats for 20 days [16]. High-MW chitosan (830 kDa) has been investigated to suppress lipid accumulation in liver and adipose tissues in a diabetic rat model for 10 weeks [6]. Gades and Stern (2003) have shown that chitosan complex (Absorbitol^®^) supplementation for 4 days can increase the fecal fat excretion in men [17]. Sugano et al. (1988) have further indicated that the effects of lowering blood cholesterol by chitosan are independent of their MW [18]. Nevertheless, Zhang et al. (2012) have indicated that the potential for hypolipidemic activity by low-MW chitosan (39.8 kDa) is higher than that of high-MW chitosan (712.6 kDa) in HF diet-fed rats for 8 weeks; but the increase in fecal fat and cholesterol excretion by high-MW chitosan is better than low-MW chitosan [19]. Chitosan oligosaccharides (≤1 kDa and ≤3 kDa) have also been found to possess better lipid-lowering effects than higher MW chitosan in HF diet-fed rats for 6 weeks [20]. However, Chiu et al. (2017) have recently suggested that low-MW chitosan (80 kDa) supplementation provides a better improvement than chitosan oligosaccharide (0.719 kDa) on lipid metabolism in HF diet-fed rats for 10 weeks [5]. Yao et al. (2008) have also indicated that the effects of high-MW chitosan (1000 kDa) on lipid-lowering and fecal fat excretion-increasing are greater than low-MW chitosan (14 kDa) in a diabetic rat model for 4 weeks [11]. In the present study, we also demonstrated that high-MW chitosan (740 kDa) had a higher efficiency than low-MW chitosan (91 kDa) on the suppression of intestinal lipid absorption and the increase of hepatic fatty acid oxidation in HF diet-fed rats for 8 weeks; but there was no significant difference between low- and high-MW chitosan on the elevation in fecal TC and TC excretion. For a comparison of the effects of low- and high-MW chitosan supplementations, the differences in the intestinal absorption efficiency of chitosan and their adsorption capacity of dietary lipids may result in their different outcome on the mediation of blood and hepatic lipometabolism. 

LPL in adipose tissue plays a critical role in lipometabolism. It can hydrolyze the core triglyceride of chylomicrons and VLDL to free fatty acid and monoglycerides. Fatty acid is uptaken and re-esterified and stored in adipose tissue or used as an energy source in muscle. Gaidhu et al., (2010) have shown that the LPL activity is increased in epididymal and visceral adipose tissues, and the lipolysis rate is decreased in subcutaneous and visceral adipose tissues in HF diet (60% kcal from fat)-fed rats [21]. In the present study, we explored that high-MW, but not low-MW, chitosan supplementation significantly decreased the TG level and LPL activity and increased the lipolysis rate in the perirenal adipose tissues compared to the HF diet group. These effects of high-MW chitosan resulted in the enhancement of lipolysis and the decrease of TG storage in the adipose tissues.

MTTP, an endoplasmic reticulum (ER)-resident chaperone, can assemble chylomicrons in the enterocytes. The increase in intestinal MTTP expression can be observed in high-cholesterol and HF diet-fed inositol-requiring enzyme 1 (IRE1β)-deficient mice, leading to hyperlipidemia and fatty liver [22]. Angptl4 is known as an endogenous inhibitor of LPL. Angptl4-deficient mice have been found to increase intestinal pancreatic lipase (PL) activity and lipid accumulation and reduce lipid excretion in feces, leading to increased weight gain and fat mass [23]. Therefore, MTTP and Angptl4 play a critical role in intestinal lipid digestion and absorption. In the present study, the increased MTTP and decreased Angptl4 protein expressions were exhibited in the intestines of HF diet-fed rats. Feeding both low- and high-MW chitosan could significantly inhibit the effects of HF diet on MTTP and Angptl4, leading to the suppression of dietary TG hydrolysis and the absorption and increase of fecal lipid excretion. High-MW chitosan was more effective than low-MW chitosan in inhibiting MTTP protein expression, indicating that high-MW chitosan may have higher potential in reducing dietary lipid absorption and increasing lipid excretion than low-MW chitosan in the intestine. However, there was no significant difference on the hypolipidemic effect and the increased fecal lipid excretion in HF diet-fed rats between high- and low-MW chitosan supplementations, suggesting chitosan may possess multiple molecular targets in intestinal lipid metabolism.

AMPK activation plays a critical role in the mediation and maintenance of cell homeostasis [24]. Increasing AMPK activation has been shown to improve NAFLD through the inhibition of de novo lipogenesis, the increase of hepatic fatty acid oxidation, and the enhancement of mitochondrial function and integrity in adipose tissue [25]. When AMPK is activated, the adipogenic transcription factors (eg. SREBPs and PPARγ) are downregulated to deactivate transcriptional activity, which impairs lipid synthesis in the liver and improves hepatic lipid metabolism. SREBP-1c is known to be involved in the synthesis of TG and fatty acid; SREBP-2 can activate cholesterol synthesis in the liver [26]. AMPK could also regulate liver PPARα activity [27]. PPARα regulates the activities of fatty acid oxidation systems including microsomal ω-oxidation and peroxisomal and mitochondrial β-oxidation that are involved in energy expenditure [28]. In PPARα-deficient *ob/ob* mice, the inhibition of fatty acid oxidation contributed to increasing weight gain and severe fatty liver [29]. The deterioration of mitochondrial fatty acid β-oxidation capacity has been suggested to cause hepatic diacylglycerol accumulation and insulin resistance [30]. In the present study, HF diet inhibited the AMPKα phosphorylation and PPARα protein expression and accelerated the protein expressions of SREBP1c, SREBP2, and PPARγ, and increased the TC and TG synthesis in the livers, which can be exhibited by a significant reversed effect by both high- and low-MW chitosan supplementations. High-MW chitosan was more efficient than low-MW chitosan on the upregulation of AMPKα phosphorylation and PPARα protein expression. 

We found that both low- and high-MW chitosan supplementations significantly prolonged the length of the small intestine. Since chitosan could significantly reduce the digestion and absorption of lipids, it was speculated that the animals may adapt to increase the length of the small intestine to increase the absorption interface for obtaining the sufficient nutrients. High-MW chitosan significantly prolonged the relative small intestine length compared to low-MW chitosan, probably because high-MW chitosan had higher adsorption capacity for dietary lipids in the small intestine than low-MW chitosan [19]. However, the mechanism of increased intestinal length by chitosan remains to be clarified in the future.

Kuipers et al. (1997) have demonstrated that apolipoprotein E (ApoE) deficiency impairs hepatic very low density lipoprotein-triglyceride (VLDL-TG) assembly and secretion in mice [31]. Maugeais et al. (2000) have found that the secretion of hepatic VLDL-TG can be promoted by ApoE expression through the increase in the production rate of VLDL-ApoB in a mouse model [32]. The patients of an ApoB-defective genetic form of familial hypobetalipoproteinemia frequently exerted fatty liver [33]. MTTP has been shown to increase hepatic VLDL-TG assembly and secretion under ApoB100 background in *ob/ob* mice [34]. In the present study, feeding HF diet inhibited the protein expressions of ApoE and MTTP in the liver, resulting in liver lipid accumulation, which could be reversed by low- and high-MW chitosan supplementation. High-MW chitosan was more efficient than low-MW chitosan on the upregulation of apoE and MTTP protein expression.

Hussain et al. (2011) have reviewed that mice possess two isoforms of MTTP, but humans express one MTTP isoform. High-fat diet was reported to stimulate the hepatic MTTP expression in rodents [35]. On the other hand, Koo (2013) has reviewed that hepatic steatosis can be observed in patients with ApoB100 mutation (hypobetalipoproteinemia) and MTTP mutation (abetalipoproteinemia) [36]. It was speculated that severe fatty liver may cause the decrease in ApoB100 and MTTP and reduce the liver secretion of VLDL-TG, leading to hepatic lipid accumulation. In our experiment condition, high-fat diet feeding induced obvious fatty liver and inhibited hepatic ApoE and MTTP protein expressions, resulting in liver lipid accumulation. However, the issue of contradictory results of feeding high-fat diet on MTTP (up-regulation in the intestine and down-regulation) in rat livers still needs more clarification and further experiments.

In this study, we found that the relative adipose weigh in the NC group was significantly higher than that in the HF group. A previous study has indicated that insulin resistance is usually presented in individuals with NAFLD, in which insulin cannot inhibit the lipolysis rate of white adipose tissue, allowing large amounts of free fatty acids entry into the liver [37]. In NAFLD patients, the secretion rate of VLDL-TG cannot be further increased when the TG infiltration in the liver exceeds 10% [38]. It was speculated that fat infiltration in the liver decreased the ability to secrete VLDL-TG, which further decreased the source of TG in the adipose tissue, leading to the decrease in the weight of adipose tissue.

The intestinal chylomicron and liver VLDL secretion, as well as the utilization of blood triglyceride by muscle, heart and adipose tissues influences the changes of blood TG levels. The previous studies have observed the decreased circulating TG levels in HF diet-fed mice [39,40]. It may be due to the reduced dietary carbohydrates. Chiu et al. (2015) have also observed the decreased blood TG levels in HF diet-fed rats that could be reversed by high-MW chitosan supplementation [41]. In the present study, we explored that HF diet feeding inhibited blood Angptl4 and hepatic ApoE and MTTP protein expressions and decreased the blood TG levels, which could be reversed by both high- and low-MW chitosan supplementations. These results indicate that both low- and high-MW chitosan are capable of ameliorating the alteration in lipid metabolism induced by high-fat diet feeding.

## 4. Materials and Methods

### 4.1. Materials

High-MW chitosan prepared from crab shell chitin was supplied from Charming & Beauty Co. (Taipei, Taiwan). The high-MW chitosan (MW: 740 kDa) was used to prepare the low-MW chitosan (MW: 91 kDa) as described previously [42]. Fourier transform infrared spectroscopy and the high-performance liquid chromatography were used to detect the deacetylation degree and the average molecular weight, respectively. The measurement of viscosity was performed by a Haake viscometer (CV20; Haake Mess-Technik GmbHu, Karlsruhe, Germany).

### 4.2. Animals and Diets

Male Sprague-Dawley rats (6-week old) were purchased from BioLASCO (Taipei, Taiwan) and acclimatized with a chow diet (Rodent Laboratory Chow, Ralston Purina, St. Louis, MO, USA) for one week. Rats were divided into four groups (*n* = 8 of each group): (1) normal control (NC) group, (2) high-fat (HF) diet group, (3) HF diet +5% high-MW (740 kDa) chitosan (HC) group, (4) HF diet +5% low-MW (91 kDa) chitosan (LC) group. The diet formulation was shown in Table 5. Rats were individually housed in stainless-steel cages under temperature (23 ± 1 °C), light (12 h light/dark cycle), and humidity (40–60% relative humidity) and were fed *ad libitum*. The dose of 5% for chitosan was selected according to the findings in the literatures [5,7,19] and our pilot study. After 8 weeks of experimental intervention, rats were fasted for 12 h and then sacrificed by exsanguination under anesthesia. Blood samples were collected for biochemical analysis. The liver, adipose, and intestine tissues (collected from duodenum to lieum) were isolated, weighted, and stored at −80 °C until the analysis for lipid profile. Feces were harvested for 3 consecutive days prior to sacrifice and stored at −80 °C until the analysis for fecal lipid contents. The animal experimental procedures were performed in accordance with the guidelines for the care and use of laboratory animals [43] and were approved by the Animal House Management Committee of the National Taiwan Ocean University.

### 4.3. Measurement of Triglyceride (TG), Cholesterol (TC), Lipoproteins, and Activities of Aspartate Aminotransferase (AST) and Alanine Aminotransferase (ALT)

The TG and TC levels in samples from liver, blood, adipose tissues and feces were measured by using Audit Diagnostics Enzymatic Assay kits (Audit Diagnostics, Cork, Ireland). The plasma low-density lipoprotein (LDL), lipoproteins {high-density lipoprotein (HDL) and very-low-density lipoprotein (VLDL)} were isolated through a density gradient performed by an ultracentrifuge (Hitachi, Tokyo, Japan) with 194,000× g at 10 °C for 3 h. The levels of AST and ALT were determined by the Randox® AST and ALT kits (Randox, Antrim, UK). The change of absorbance at 340 nm was detected by a spectrophotometer (U-2880A; Hitachi, Tokyo, Japan).

### 4.4. Measurement of Lipolysis Rate 

Lipolysis rate was measured as described previously [41]. Briefly, 0.2 g adipose tissues were minced and added into a N-tris-(hydroxymethyl)-methyl-2-aminoethanesulfonic acid (25 mM) buffer containing isoproterenol (1 μM). A glycerol detection kit (Randox, Amtrim, UK) was used to measure the glycerol levels in samples after 1, 2, and 3 h of incubation at 37 °C and the absorbance was recorded at 520 nm. The lipolysis rate was expressed by nano-moles glycerol/gram adipose tissue/h.

### 4.5. Measurement of Lipoprotein Lipase (LPL) Activity

LPL activity was determined as described previously [41]. The 0.1 g adipose tissues were minced and added into a Krebs-Ringer bicarbonate buffer (pH 7.4) containing 10 units/mL heparin for 60 min at 37 °C, and then samples were incubated with equal volume of *p*-nitrophenyl butyrate (2 mM). LPL activity was measured by the levels of *p*-nitrophenol formation over 10 min incubation. The absorbance was detected at 400 nm.

### 4.6. Histological Analysis of Liver

Five micrometer thick hepatic paraffin sections were used for histological examination. The hematoxylin and eosin (H&E)-stained tissue sections were observed and imaged by a photo microscope (Nikon Eclipse TS100, Nikon Instruments, Melville, NY, USA) equipped with a digital camera (Nikon D5100, Nikon Instruments, Melville, NY, USA).

### 4.7. Protein Expression Analysis 

The protein expression was measured by Western blotting as described previously [44]. The equal protein extracts were spiked into 8%–12% sodium dodecyl sulfate-polyacrylamide electrophoresis (SDS-PAGE) gel, and then transferred to polyvinylidene difluoride (PVDF) membranes (Bio-Rad, Hercules, CA, USA). The membranes were blocked for 1 h, and then incubated with primary antibodies including AMPKα and phosphorylated AMPKα (p-AMPKα) (Cell Signaling Technology, Danvers, MA, USA), Angptl4, PPAR-γ, SREBP1c, PPAR-α, MTTP, SREBP2, β-actin (Santa Cruz Biotechnology, Santa Cruz, CA, USA), and ApoE (Bioss Antibodies, Woburn, MA, USA) overnight. Next, the membranes were hybridized with secondary antibodies. The antigen-antibody complexes were visualized by using Bio-Rad enhanced chemiluminescence kit and exposed to Fujifilm X-ray film (Fujifilm, Tokyo, Japan). The protein bands were densitometrically analyzed with an image software (Image J 1.51; National Institutes of Health, Bethesda, MD, USA).

### 4.8. Statistical Analysis

All results are presented as the Mean ± Standard Deviation (SD). The difference among experimental groups is assessed by one-way analysis of variance (ANOVA) followed by Dunnett's test with the IBM SPSS Statistics 22.0 software (International Business Machines Corporation, Armonk, NY, USA).

## 5. Conclusions

In conclusion, the present study showed that in a HF diet-fed rat model, both high- and low MW chitosan (1) inhibit MTTP and increase Angptl4 protein expressions in the intestine and increase the small intestine length and fecal lipid excretion; (2) activate AMPK and inhibit downstream lipogenesis transcription factors (SREBP2, SREBP1c, and PPARγ) and protein expressions, and promote PPARα protein expression in the liver; (3) and promote liver VLDL secretion-related proteins (ApoE and MTTP) expressions, leading in reducing fatty synthesis, increasing β-oxidation, and improving fatty liver. Comparison of low- and high-MW chitosan, high-MW chitosan is more effective than low-MW chitosan on the regulation of liver weight, perirenal adipose weight, relative small intestine length, adipose TG level and LPL activity, intestinal MTTP expression, hepatic phospho-AMPKα, PPARα, apoE, and MTTP expression.

## Figures and Tables

**Table 1:** The changes of body weight, liver and adipose tissue weight, and small intestine length in rats fed with different experimental diets for 8 weeks.

Diet	NC	HF	HC	LC
Food intake (g)	27.9 ± 1.7 ^a^	25.3 ± 1.9 ^a^	26.7 ± 1.3 ^a^	25.9 ± 1.5 ^a^
Caloric intake (kcal/kg/day)	107.0± 6.4 ^a^	109.3 ± 8.3 ^a^	115.0 ± 5.8 ^a^	111.9 ± 6.5 ^a^
Body weight (g)	539.9 ± 22.5 ^a^	578 ± 47.3 ^b^	518.3 ± 34.1 ^a^	543.4 ± 18.9 ^a^
Liver weight (g)	14.5 ± 1.5 ^a^	33.3 ± 4.9 ^b^	20.0 ± 1.9 ^c^	23.0 ± 3.2 ^d^
Relative liver weight (g/100 g BW)	2.7 ± 0.2 ^a^	5.9 ± 0.7 ^b^	3.9 ± 0.4 ^a^	4.2 ± 0.5 ^a^
Adipose tissue weight (g)	28.7 ± 6.3 ^a,b^	23.1 ± 5.2 ^b^	18.0 ± 5.6 ^b,c^	20.7 ± 3.1 ^b,c^
Relative adipose weight (g/100 g BW)	5.2 ± 1.0 ^a^	4.1 ± 0.8 ^b^	3.5 ± 1.0 ^b^	3.8 ± 0.5 ^b^
Perirenal adipose weight (g)	16.7 ± 3.6 ^a^	14.0 ± 3.4 ^a^	10.7 ± 2.7 ^b^	12.1 ± 2.2 ^a^
Epididymal adipose weight (g)	12.0 ± 3.0 ^a^	9.1 ± 2.0 ^b^	7.3 ± 3.3 ^b^	8.6 ± 1.5 ^b^
Small intestine length (cm)	116.3 ± 2.6 ^a,b^	116.0 ± 5.2 ^a^	132.6 ± 11.4 ^b,c^	129.1 ± 10.3 ^b,c^
Relative small intestine length (cm/100 g BW)	21.4 ± 1.2 ^a^	20.7 ± 1.6 ^a^	25.6 ± 1.4 ^b^	23.5 ± 1.9 ^c^

Data are presented as mean ± standard deviation (SD) for each group (*n* = 7–8). Different letters (a, b, and c) indicate significant differences (*p* < 0.05). NC: normal control +5% cellulose; HF: High-fat diet +5% cellulose; HC: High-fat diet +5% High molecular weight chitosan; LC: High-fat diet +5% Low molecular weight chitosan.

**Table 2:** The changes of plasma lipids, TNF-α, and liver functional markers levels in rats fed with different experimental diets for 8 weeks.

Diet	NC	HF	HC	LC
Total cholesterol (mg/dL)	63.1 ± 9.3 ^a^	87.4 ± 11.7 ^b^	56.2 ± 7.9 ^a^	52.3 ± 17.0 ^a^
HDL-C (mg/dL)	44.7 ± 5.8 ^a^	9.1 ± 7.9 ^b^	26.7 ± 2.7 ^b^	25.5 ± 4.1 ^b^
LDL-C + VLDL-C (mg/dL)	18.4 ± 7.2 ^a^	58.3 ± 12.0 ^b^	29.5 ± 10.2 ^c^	26.7 ± 18.7 ^a,c^
VLDL-C (mg/dL)	14.9 ± 8.1 ^a^	28.8 ± 8.2 ^b^	16.0 ± 6.4 ^a^	14.0 ± 12.7 ^a^
LDL-C (mg/dL)	3.5 ± 1.6 ^a^	29.5 ± 11.4 ^b^	13.6 ± 7.7 ^c^	12.7 ± 10.5 ^c^
TC/HDL-C (mg/dL)	1.4 ± 0.2 ^a^	3.2 ± 0.9 ^b^	2.1 ± 0.5 ^c^	2.1 ± 0.9 ^a,c^
HDL-C/LDL-C + VLDL-C ratio	2.8 ± 1.2 ^a^	0.5 ± 0.2 ^b^	1.0 ± 0.4 ^c^	2.2 ± 2.7 ^a,b,c^
Triglyceride (mg/dL)	96.2 ± 43.3 ^a^	34.5 ± 5.1 ^b^	44.1 ± 8.8 ^c^	45.8 ± 13.0 ^c^
TNF-α (pg/dL)	10.9 ± 2.3 ^a^	36.8 ± 13.7 ^b^	17.1 ± 3.6 ^c^	22.8 ± 7.3 ^c^
ALT (U/L)	15.7 ± 3.5 ^a^	72.2 ± 32.1 ^b^	25.5 ± 15.9 ^a,c^	35.1 ± 25.0 ^c^
AST (U/L)	42.1 ± 16.6 ^a^	79.1 ± 42.8 ^b^	39.4 ± 22.2 ^a^	59.7 ± 31.2 ^a,b^

Data are presented as mean ± SD for each group (*n* = 7–8). Different letters (a, b, and c) indicate significant differences (*p* < 0.05). NC: normal control + 5% cellulose; HF: High-fat diet + 5% cellulose; HC: High-fat diet + 5% High molecular weight chitosan; LC: High-fat diet + 5% Low molecular weight chitosan. ALT = alanine aminotransferase; AST = aspartate aminotransferase

**Table 3:** The changes of hepatic cholesterol and triglyceride levels in rats fed with different experimental diets for 8 weeks.

Diet	NC	HF	HC	LC
Total cholesterol	-	-	-	-
(mg/g liver)	2.7 ± 1.0 ^a^	152.6 ± 13.7 ^b^	52.6 ± 28.4 ^c^	65.4 ± 33.2 ^c^
(g/liver)	0.04 ± 0.02 ^a^	5.1 ± 0.8 ^b^	1.1 ± 0.6 ^c^	1.6 ± 0.9 ^c^
Triglyceride	-	-	-	-
(mg/g liver)	12.1 ± 5.0 ^a^	96.0 ± 22.2 ^b^	49.2 ± 20.1 ^c^	58.7 ± 29.0 ^c^
(g/liver)	0.2 ± 0.1 ^a^	3.1 ± 0.5 ^b^	1.0 ± 0.4 ^c^	1.4 ± 0.8 ^c^

Data are presented as mean ± SD for each group (*n* = 7–8). Different letters (a, b, and c) indicate significant differences (*p* < 0.05). NC: normal control +5% cellulose; HF: High-fat diet +5% cellulose; HC: High-fat diet +5% High molecular weight chitosan; LC: High-fat diet +5% Low molecular weight chitosan.

**Table 4:** The changes of fecal weight, total cholesterol and triglyceride concentration in rats fed with different experimental diets for 8 weeks.

Diet	NC	HF	HC	LC
Feces wet weight (g/day)	2.9 ± 0.2 ^a^	2.8 ± 0.2 ^a^	3.1 ± 0.5 ^b^	3.6 ± 0.5 ^b^
Feces dry weight (g/day)	2.2 ± 0.2 ^a,b^	2.0 ± 0.2 ^b^	2.4 ± 0.5 ^a^	2.9 ± 0.5 ^a,c^
Total cholesterol	-	-	-	-
(mg/g feces)	2.4 ± 0.4 ^a^	12.2 ± 1.5 ^b^	24.2 ± 4.8 ^c^	23.2 ± 4.7 ^c^
(mg/day)	5.4 ± 1.1 ^a^	24.3 ± 3.4 ^b^	59.4 ± 20 ^c^	66.5 ± 17.3 ^c^
Triglyceride	-	-	-	-
(mg/g feces)	0.4 ± 0.2 ^a^	0.7 ± 0.2 ^b^	1.8 ± 0.4 ^c^	2.0 ± 0.3 ^c^
(mg/day)	0.9 ± 0.4 ^a^	1.4 ± 0.5 ^b^	4.5 ± 1.5 ^c^	5.7 ± 1.4 ^c^

Data are presented as mean ± SD for each group (*n* = 7–8). Different letters indicate significant differences (*p* < 0.05). NC: normal control +5% cellulose; HF: High-fat diet + 5% cellulose; HC: High-fat diet + 5% High molecular weight chitosan; LC: High-fat diet + 5% Low molecular weight chitosan.

**Table 5:** Composition of experimental diets (%).

Ingredient (%)	NC	HF	HC	LC
Casein	20	20	20	20
Lard	3	13	13	13
Soybean oil	2	2	2	2
Vitamin mixture ^1^	1	1	1	1
Minerals ^2^	4	4	4	4
Cholesterol	-	0.5	0.5	0.5
Choline chloride	0.2	0.2	0.2	0.2
Cholic acid	-	0.2	0.2	0.2
Corn starch	64.8	54.1	54.1	54.1
Cellulose	5	5	-	-
High molecular weight chitosan ^3^	-	-	5	-
Low molecular weight chitosan ^4^	-	-	-	5

NC: normal control (3% Lard +2% soybean oil) +5% cellulose; HF: High-fat diet (13% Lard +2% Soybean oil) +5% cellulose; HC: High-fat diet +5% High molecular weight chitosan; LC: High-fat diet +5% Low molecular weight chitosan. ^1^ AIN-93 vitamin mixture; ^2^ AIN-93 mineral mixture; ^3^ The average MW and DD of High molecular weight chitosan about 7.4 × 10^5^ Dalton and 91%, respectively; ^4^ The average MW and DD of Low molecular weight chitosan about 9.1 × 10^4^ Dalton and 92%, respectively.
